# Thyrotoxic Periodic Paralysis: Case Presentation With Tetraparesis and Cardiac Dysrhythmia

**DOI:** 10.7759/cureus.29759

**Published:** 2022-09-29

**Authors:** Ayodeji Dosu, Mehak Gupta, Olivia Walsh, Jayesh Makan

**Affiliations:** 1 Internal Medicine, The Shrewsbury and Telford Hospital National Health Service (NHS) Trust, Telford, GBR; 2 Cardiology, The Shrewsbury and Telford Hospital National Health Service (NHS) Trust, Telford, GBR

**Keywords:** dysrhythmia, caucasian, thyroid, hypokalaemia, cardiac, thyrotoxic, paralysis, periodic, thpp

## Abstract

Thyrotoxic hypokalaemic periodic paralysis (THPP) is a rare complication of hyperthyroidism that is potentially life-threatening if not treated promptly. It is more common in Asian and Polynesian populations and very few cases have been reported to date in people of White ethnicity. We present a case report of a young male patient of White ethnicity, who was initially brought in as a stroke alert with tetraparesis which was ruled out on initial assessment, but then had a syncopal episode and was noted to be initially bradycardic and subsequently tachycardic. Blood tests showed hypokalaemia and hypophosphataemia and he was treated as a hypokalaemic periodic paralysis patient. Intravenous potassium replacement was commenced. Symptoms and ECG changes resolved with correction of potassium levels. Thyroid function tests requested later were suggestive of hyperthyroidism and the diagnosis of thyrotoxic hypokalaemic periodic paralysis was made. This is an interesting case given its rarity, and this case report highlights the importance of early diagnosis and prompt treatment.

## Introduction

Hypokalaemic periodic paralysis is a rare life-threatening condition of which there are two main subtypes; familial hypokalaemic periodic paralysis (FHPP) and thyrotoxic hypokalaemic periodic paralysis (THPP). FHPP is more common in western countries and paediatric patients while THPP is more common in Asian and Polynesian populations. It occurs more commonly in age groups of 20-40 and the male to female ratio is 20:1. Presentation ranges from transient limb weakness [1] to myalgia and limb paralysis [2]. This case report denotes the importance of the consideration of a diagnosis of THPP in patients presenting to the emergency department with acute muscle weakness and syncope. It is a reversible condition if recognised and treated promptly.

## Case presentation

A 22-year-old gentleman with a background of coeliac disease was admitted to the hospital with generalized weakness, predominantly in his lower limbs. He was brought to the emergency department as a stroke alert for thrombolysis but this diagnosis was excluded after the initial assessment. He had woken up with these symptoms and admitted to smoking cannabis before sleeping. He denied binge eating but reported nausea and as a result, had poor oral intake. He had an episode of vomiting prior to presenting to the hospital but no diarrhoea. There was a vague family history of thyroid disease, possibly hypothyroidism.

On examination, he was noted to have flaccid paralysis, with power grade 3/5 in upper limbs and grade 1/5 in lower limbs with both proximal and distal muscles being affected equally. The rest of the neurological and systemic examinations were normal.

**Figure 1:**
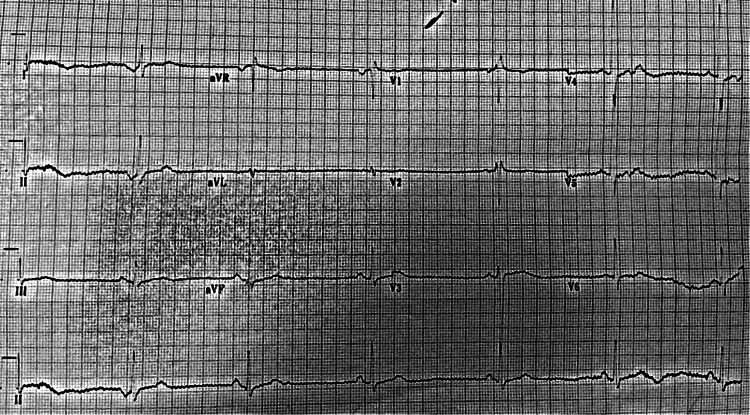
Sinus bradycardia with incomplete right bundle branch block

**Figure 2:**
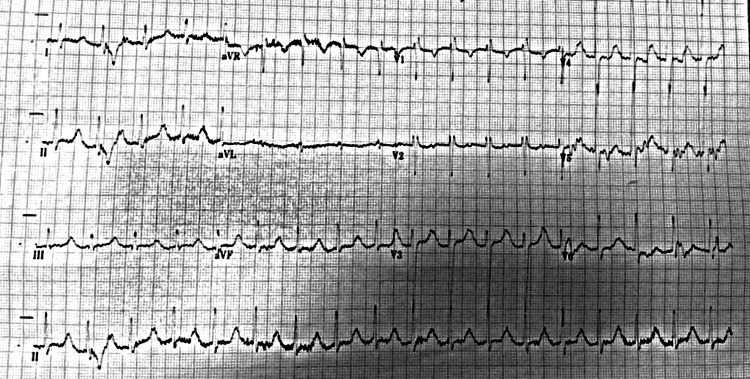
ECG showing accelerated junctional rhythm, a corrected QT interval of 614ms and an incomplete right bundle branch block.

Whilst in the department he had a syncopal episode and an electrocardiogram (ECG) showed marked sinus bradycardia (35 beats per minute) with an incomplete right bundle branch block (Figure 1). A dose of 500 micrograms of atropine was administered intravenously, and he was put on a cardiac monitor. After that, ECG revealed accelerated junctional rhythm, a corrected QT interval of 614ms and an incomplete right bundle branch block (Figure 2). Venous blood gas showed potassium levels of 1.8mmol/l, later confirmed with laboratory investigation. He was also noted to have hypophosphataemia - 0.58mmol/l (normal range 0.7mmol/L - 1.0mmol/L) and abnormal thyroid function test, suggestive of overactive thyroid - thyroid-stimulating hormone (TSH) < 0.01mu/L (normal 0.30mu/L - 4.20mu/L); free triiodothyronine (FT3) -33.5pmol/L (normal 3.1pmol/L - 6.8pmol/L), free thyroxine (FT4) - 65.8pmol/L (normal 11.0pmol/L - 22.0pmol/L). The remaining blood tests including renal function, liver function, and full blood count were within normal limits. CT head revealed no acute intracranial abnormality.

**Figure 3:**
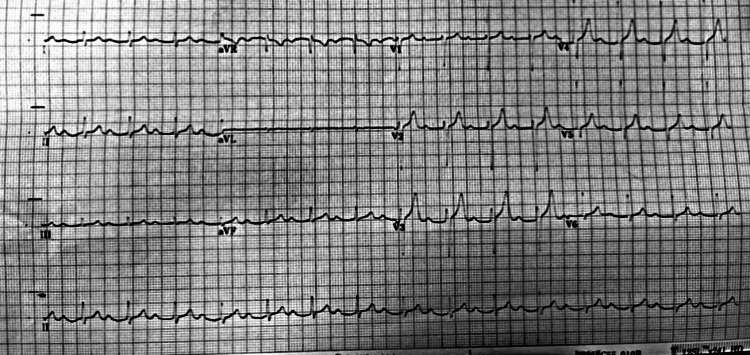
ECG showing normal sinus rhythm with a first-degree atrioventricular block

A diagnosis of hypokalaemic periodic paralysis was made. He was started on intravenous (IV) potassium replacement as well as IV Magnesium due to his prolonged QT interval, and was transferred to the coronary care unit for cardiac monitoring. His symptoms resolved completely 12 hours post admission as his potassium levels were normalised. An interesting thing to note was that most of his ECG changes resolved post potassium level correction except he now had a first-degree atrioventricular block (Figure 3).

Following the resolution of his symptoms, an endocrinology review was sought and a diagnosis of THPP was made. He was started on carbimazole and an outpatient endocrinology review was arranged. The patient was subsequently discharged from the hospital. He was reviewed in the endocrinology clinic and has remained symptom-free with normal potassium levels. He will be considered for thyroidectomy after he becomes euthyroid.

## Discussion

Hypokalaemia in THPP is attributed to a shift of potassium into the cells, particularly muscle cells, rather than due to potassium loss. It is hypothesised that this potassium shift is a result of increased activity of sodium-potassium-adenosine-triphosphate (NA+K+ATPase) pumps driven by elevated levels of T3 (the most active thyroid hormone [3]. The severity of hypokalaemia often correlates with the severity of paralysis. Hypophosphataemia and mild hypomagnesaemia are also often present in THPP [4].

There have been studies incorporating a genetic susceptibility and a mutation in the gene *KCNJ2* which points towards a familial inheritance form of hypokalaemic periodic paralysis [5]. Mutations in the gene *KCNJ18* which codes for the potassium channel Kir2.6 have also been reported in recent studies. Kir2.6 is thought to be a contributor to cell membrane excitability and is primarily expressed in skeletal muscles [6]. An increased rate of ion channel transcription in the presence of high thyroid hormones is hypothesised as driving the accumulation of potassium within cells [7].

There have been many case reports identifying repeated/recurrent attacks of this disorder. Establishing normal potassium levels between attacks is an important way of distinguishing THPP from conditions such as familial hypokalaemic periodic paralysis, proximal myopathy, proximal renal tubular acidosis, profuse diarrhoea, Bartter’s and Gitelman’s syndrome.

Patients are generally young males between the ages of 20 and 40 [8]. THPP is most common in patients of Polynesian ethnicity and Asian ethnicity, with a few cases (5%) reported in Caucasians [9]. The patient presented in our case is Caucasian. Proximal muscle groups are seemingly more affected than distal groups [10]. Rarely, there can be diaphragmatic involvement leading to respiratory compromise [11,12]. Episodes classically begin on waking, following strenuous physical activity, or after consumption of a carbohydrate-heavy meal [3]. This patient also developed symptoms on waking up but had reported poor oral intake. He also admitted to smoking cannabis prior to the onset of symptoms. Binge eating after marijuana leading to hypokalaemic periodic paralysis has been reported previously [13], but whether there is any clear association between smoking marijuana alone and THPP needs to be further investigated.

The prompt identification and diagnosis of THPP are extremely important to enable the formulation of optimal management plans for these patients. In the acute setting, intravenous potassium replacement is the most important intervention. However, vigilance should be maintained by monitoring for rebound hyperkalaemia [12]. Potassium replacement is not a long-term solution for these patients, as in between attacks, their serum potassium levels will most likely normalise. The definitive treatment for these patients is the management of the underlying cause, in this case, hyperthyroidism [14,15].

## Conclusions

THPP should be suspected in young patients presenting with acute muscle weakness and severe hypokalaemia. Though, more common in Asian and Polynesian populations, a few cases have been recognised in Caucasian population. It is of utmost importance to promptly diagnose this condition as unlike many other forms of periodic paralysis, it can be treated relatively easily with successful management of hyperthyroidism. Various dysrhythmias can be noted but most of these will resolve with normalisation of potassium.
